# Supplementary material for: Consolidation alters motor sequence-specific distributed representations
Source: eLife. 2019 Mar 18;8:e39324. doi: 10.7554/eLife.39324 (PMC6461441; doi:10.7554/eLife.39324)
Supplement: Supplementary file 1. [file elife-39324-supp1.docx]

mean_seq_duration ~ seq_new * blocks + (blocks+sequences | participants)
==========================================================================================
Model: MixedLM Dependent Variable: mean_seq_duration
No. Observations: 1146 Method: REML
No. Groups: 18 Scale: 0.0368
Min. group size: 62 Likelihood: 165.9658
Max. group size: 64 Converged: Yes
Mean group size: 63.7
------------------------------------------------------------------------------------------
 Coef. Std.Err. z P>|z| [0.025 0.975]
------------------------------------------------------------------------------------------
Intercept 1.269 0.076 16.790 0.000 1.121 1.417
New Sequences 0.365 0.047 7.776 0.000 0.273 0.457
Rate of speed changes/block -0.006 0.005 -1.304 0.192 -0.016 0.003
Rate of speed changes/block: New Sequences -0.018 0.002 -7.403 0.000 -0.023 -0.013
==========================================================================================
