# Supplementary material for: Consolidation alters motor sequence-specific distributed representations
Source: eLife. 2019 Mar 18;8:e39324. doi: 10.7554/eLife.39324 (PMC6461441; doi:10.7554/eLife.39324)
Supplement: Supplementary file 2. [file elife-39324-supp2.docx]

num_correct_seq ~ seq_new * blocks + (blocks+sequences | participants)
==========================================================================================
Model: MixedLM Dependent Variable: num_correct_seq
No. Observations: 1152 Method: REML
No. Groups: 18 Scale: 0.6018
Min. group size: 64 Likelihood: -1409.7169
Max. group size: 64 Converged: No
Mean group size: 64.0
------------------------------------------------------------------------------------------
 Coef. Std.Err. z P>|z| [0.025 0.975]
------------------------------------------------------------------------------------------
Intercept 4.691 0.079 59.215 0.000 4.536 4.846
New Sequences -0.304 0.101 -3.003 0.003 -0.503 -0.106
Rate accuracy change/block -0.006 0.057 -0.101 0.919 -0.117 0.106
Rate accuracy change/block: New Sequences 0.014 0.010 1.434 0.152 -0.005 0.034
==========================================================================================
