# Supplementary material for: Consolidation alters motor sequence-specific distributed representations
Source: eLife. 2019 Mar 18;8:e39324. doi: 10.7554/eLife.39324 (PMC6461441; doi:10.7554/eLife.39324)
Supplement: Supplementary file 3. [file elife-39324-supp3.docx]

mean_seq_duration ~ sequences*blocks + (1|participants)

==========================================================================================

Model: MixedLM Dependent Variable: mean_seq_duration

No. Observations: 571 Method: REML

No. Groups: 18 Scale: 0.0655

Min. group size: 30 Likelihood: -76.5056

Max. group size: 32 Converged: Yes

Mean group size: 31.7

------------------------------------------------------------------------------------------

Coef. Std.Err. z P>|z| [0.025 0.975]

------------------------------------------------------------------------------------------

Intercept 1.630 0.071 22.931 0.000 1.490 1.769

New Sequence 2 0.025 0.045 0.558 0.577 -0.063 0.113

Rate of speed changes/block -0.023 0.003 -7.157 0.000 -0.030 -0.017

Rate of speed changes/block: New Sequence 2 -0.005 0.005 -1.174 0.241 -0.015 0.004

==========================================================================================

num_correct_seq ~ sequences*blocks + (1|participants)

==========================================================================================

Model: MixedLM Dependent Variable: num_correct_seq

No. Observations: 571 Method: REML

No. Groups: 18 Scale: 0.6209

Min. group size: 30 Likelihood: -689.3501

Max. group size: 32 Converged: Yes

Mean group size: 31.7

------------------------------------------------------------------------------------------

Coef. Std.Err. z P>|z| [0.025 0.975]

------------------------------------------------------------------------------------------

Intercept 4.553 0.102 44.450 0.000 4.353 4.754

New Sequence 2 -0.245 0.138 -1.772 0.076 -0.517 0.026

Rate of accuracy changes/block -0.007 0.010 -0.728 0.467 -0.027 0.012

Rate of accuracy changes/block: New Sequence 2 0.028 0.014 1.936 0.053 -0.000 0.056

==========================================================================================
