# Supplementary material for: Consolidation alters motor sequence-specific distributed representations
Source: eLife. 2019 Mar 18;8:e39324. doi: 10.7554/eLife.39324 (PMC6461441; doi:10.7554/eLife.39324)
Supplement: Supplementary file 4. [file elife-39324-supp4.docx]

mean_seq_duration ~ sequences*blocks + (1|participants)
==========================================================================================
Model: MixedLM Dependent Variable: mean_seq_duration
No. Observations: 575 Method: REML
No. Groups: 18 Scale: 0.0222
Min. group size: 31 Likelihood: 226.1710
Max. group size: 32 Converged: Yes
Mean group size: 31.9
------------------------------------------------------------------------------------------
 Coef. Std.Err. z P>|z| [0.025 0.975]
------------------------------------------------------------------------------------------
Intercept 1.256 0.057 21.949 0.000 1.144 1.368
Trained Sequence 2 0.031 0.026 1.191 0.234 -0.020 0.082
Rate of speed changes/block -0.008 0.002 -4.023 0.000 -0.011 -0.004
Rate of speed changes/block: Trained Sequence 2 -0.000 0.003 -0.165 0.869 -0.006 0.005
==========================================================================================

num_correct_seq ~ sequences*blocks + (1|participants)
==========================================================================================
Model: MixedLM Dependent Variable: num_correct_seq
No. Observations: 575 Method: REML
No. Groups: 18 Scale: 0.4050
Min. group size: 31 Likelihood: -569.8356
Max. group size: 32 Converged: Yes
Mean group size: 31.9
------------------------------------------------------------------------------------------
 Coef. Std.Err. z P>|z| [0.025 0.975]
------------------------------------------------------------------------------------------
Intercept 4.694 0.081 58.093 0.000 4.535 4.852
Trained Sequence 2 -0.030 0.111 -0.267 0.789 -0.248 0.188
Rate of accuracy changes/block -0.012 0.008 -1.414 0.157 -0.028 0.004
Rate of accuracy changes/block: Trained Seq. 2 0.014 0.012 1.207 0.228 -0.009 0.036
==========================================================================================
